# Effect of Treatment of Obstructive Sleep Apnea on Depressive Symptoms: Systematic Review and Meta-Analysis

**DOI:** 10.1371/journal.pmed.1001762

**Published:** 2014-11-25

**Authors:** Marcus Povitz, Carmelle E. Bolo, Steven J. Heitman, Willis H. Tsai, JianLi Wang, Matthew T. James

**Affiliations:** 1 Department of Community Health Sciences, University of Calgary, Calgary, Alberta, Canada; 2 Department of Medicine, Western University, London, Ontario, Canada; 3 Department of Medicine, University of Calgary, Calgary, Alberta, Canada; 4 Department of Psychiatry, University of Calgary, Calgary, Alberta, Canada; University of Western Sydney, Australia

## Abstract

**Background:**

Obstructive sleep apnea (OSA) is associated with increased morbidity and mortality, and decreased quality of life. Treatment with continuous positive airway pressure (CPAP) or mandibular advancement devices (MADs) is effective for many symptoms of OSA. However, it remains controversial whether treatment with CPAP or MAD also improves depressive symptoms.

**Methods and Findings:**

We performed a systematic review and meta-analysis of randomized controlled trials that examined the effect of CPAP or MADs on depressive symptoms in patients with OSA. We searched Medline, EMBASE, the Cochrane Central Registry of Controlled Trials, and PsycINFO from the inception of the databases until August 15, 2014, for relevant articles.

In a random effects meta-analysis of 19 identified trials, CPAP treatment resulted in an improvement in depressive symptoms compared to control, but with significant heterogeneity between trials (*Q* statistic, *p*<0.001; *I* ^2^ = 71.3%, 95% CI: 54%, 82%). CPAP treatment resulted in significantly greater improvement in depressive symptoms in the two trials with a higher burden of depression at baseline (meta-regression, *p*<0.001). The pooled standardized mean difference (SMD) in depressive symptoms with CPAP treatment in these two trial populations with baseline depression was 2.004 (95% CI: 1.387, 2.621), compared to 0.197 (95% CI: 0.059, 0.334) for 15 trials of populations without depression at baseline. Pooled estimates of the treatment effect of CPAP were greater in parallel arm trials than in crossover trials (meta-regression, *p* = 0.076). Random effects meta-analysis of five trials of MADs showed a significant improvement in depressive symptoms with MADs versus controls: SMD = 0.214 (95% CI: 0.026, 0.401) without significant heterogeneity (*I* ^2^ = 0%, 95% CI: 0%, 79%). Studies were limited by the use of depressive symptom scales that have not been validated specifically in people with OSA.

**Conclusions:**

CPAP and MADs may be useful components of treatment of depressive symptoms in individuals with OSA and depression. The efficacy of CPAP and MADs compared to standard therapies for depression is unknown.

*Please see later in the article for the Editors' Summary*

## Abstract 2

In a meta-analysis of randomized controlled trials, Matthew James and colleagues investigate the effects of continuous positive airway pressure or mandibular advancement devices on depression.

*Please see later in the article for the Editors' Summary*

## Editors' Summary

**Background:**

Obstructive sleep apnea (OSA) is a sleep-related breathing disorder that is particularly common among middle-aged and elderly people, although most are unaware that they have the condition. It is characterized by the occurrence of numerous brief (ten seconds or so) breathing interruptions during sleep. These “apneas” occur when relaxation of the upper airway muscles decreases airflow, which lowers the level of oxygen in the blood. Consequently, affected individuals are frequently aroused from deep sleep as they struggle to breathe. Symptoms of OSA include loud snoring and daytime sleepiness. Treatments include lifestyle changes such as losing weight (excess fat around the neck increases airway collapse) and smoking cessation. Mild to moderate OSA can also be treated using a mandibular advancement device (MAD), a “splint” that fits inside the mouth and pushes the jaw and tongue forward to increase the space at the back of the throat and reduce airway narrowing. For severe OSA, doctors recommend continuous positive airway pressure (CPAP), in which a machine blows pressurized air into the airway through a facemask to keep it open.

**Why Was This Study Done?:**

OSA is a serious condition that is associated with an increased risk of illness and death. Clinical depression (long-lasting, overwhelming feelings of sadness and hopelessness), for example, is common among people with OSA. The interaction between these frequently co-morbid (co-existing) conditions is complex. The sleep disruption and weight gain that are often associated with depression could cause or worsen OSA. Conversely, OSA could trigger depression by causing sleep disruption and by inducing cognitive changes (changes in thinking) by intermittently starving the brain of oxygen. If the latter scenario is correct, then treating OSA with CPAP or MADs might improve depressive symptoms. Several trials have investigated this possibility, but their results have been equivocal. Here, the researchers undertake a systematic review and meta-analysis of randomized controlled trials that have examined the effect of CPAP or MADs on depressive symptoms in patients with OSA to find out whether treating co-morbid OSA in patients with depression can help to treat depression. A randomized controlled trial compares the outcomes of individuals chosen to receive different interventions through the play of chance, a systematic review uses predefined criteria to identify all the research on a given topic, and meta-analysis uses statistical methods to combine the results of several studies.

**What Did the Researchers Do and Find?:**

The researchers identified 22 trials that investigated the effects of CPAP or MAD treatment in patients with OSA and that measured depressive symptoms before and after treatment. Meta-analysis of the results of 19 trials that provided information about the effect of CPAP on depressive symptoms indicated that CPAP improved depressive symptoms compared to the control intervention (usually sham CPAP) but revealed considerable heterogeneity (variability) between trials. Notably, CPAP treatment resulted in a greater improvement in depressive symptoms in trials in which there was a high prevalence of depression at baseline than in trials in which there was a low prevalence of depression at baseline. Moreover, the magnitude of this improvement in depressive symptoms in trials with a high prevalence of depression at baseline was large enough to be clinically relevant. Meta-analysis of five trials that provided information about the effect of MADs on depressive symptoms indicated that MADs also improved depressive symptoms compared to the control intervention (sham MAD).

**What Do These Findings Mean?:**

These findings suggest that both CPAP and MAD treatment for OSA can result in modest improvements in depressive symptoms and that populations with high initial levels of depressive symptoms may reap the greatest benefits of CPAP treatment. These findings give no indication of the efficacy of CPAP and MADs compared to standard treatments for depression such as antidepressant medications. Moreover, their accuracy may be limited by methodological limitations within the trials included in the meta-analyses reported here. For example, the questionnaires used to measure depression in these trials were not validated for use in people with OSA. Further high-quality randomized controlled trials are therefore needed to confirm the findings of this systematic review and meta-analysis. For now, however, these findings suggest that the use of CPAP and MADs may help improve depressive symptoms among people with OSA.

**Additional Information:**

Please access these websites via the online version of this summary at http://dx.doi.org/10.1371/journal.pmed.1001762.

- The US National Heart, Lung, and Blood Institute has information (including several videos) about sleep apnea (in English and Spanish)
- The UK National Health Service Choices website provides information and personal stories about obstructive sleep apnea and depression
- The not-for-profit American Sleep Apnea Association provides detailed information about sleep apnea for patients and healthcare professionals, including personal stories about the condition
- The US National Institute of Mental Health provides information on all aspects of depression (in English and Spanish)
- The Anxiety and Depression Association of America provides information about sleep disorders
- The MedlinePlus encyclopedia has a page on obstructive sleep apnea; MedlinePlus provides links to further information and advice about obstructive sleep apnea and about depression (in English and Spanish)

## Introduction

Obstructive sleep apnea (OSA), characterized by repetitive upper airway obstruction during sleep, is associated with increased morbidity and mortality and decreased quality of life [1],[2]. OSA has multiple mechanisms that ultimately lead to airflow limitation, intermittent hypoxia, and arousal from sleep [3]. OSA is associated with poor-quality sleep, insomnia, and neurocognitive symptoms of poor memory and irritability [4]. Poor sleep, fatigue, and cognitive impairment are also common in depression. Depression is highly prevalent in individuals with OSA, with some studies reporting that up to 63% of individuals are affected [5]. Previous studies have attempted to quantify the strength of the associations between excessive sleepiness, depression, and OSA, and have suggested that depression, rather than OSA, may be more strongly associated with excessive sleepiness [6]. OSA may cause depression on the basis of sleep loss, sleep disruption, and cognitive changes induced by intermittent hypoxemia, while weight gain and sleep disruption due to depression could cause or worsen OSA [5].

Treatment with continuous positive airway pressure (CPAP) is effective for all degrees of OSA [7], and has been shown to reduce excessive sleepiness as well as adverse events related to other medical conditions [8]–[10]. Commonly used and effective alternatives to CPAP in individuals with mild to moderate OSA are mandibular advancement devices (MADs). These devices, though less effective than CPAP, are better tolerated by many individuals with OSA and may offer similar reductions in sleepiness [11]. However, it remains controversial whether treatment with CPAP or MADs also improves depressive symptoms.

The effect of treatment of sleep apnea on depressive symptoms has been evaluated in a number of trials; however, results have been equivocal [7]. The last systematic review that addressed this question concluded that the effects of OSA treatment on depressive symptoms were limited and inconsistent between trials because of a high level of heterogeneity. However, this previous review was limited to studies of CPAP that used the Hospital Anxiety and Depression Scale (HADS). Therefore, the objective of the present systematic review was to comprehensively examine the effects of CPAP and MADs on depressive symptoms across various depression questionnaires in adults with OSA. We hypothesized that the study design (i.e., use of crossover versus parallel arm design), choice of depression scale, baseline prevalence of depression in the trial population, and treatment duration would explain the previously reported heterogeneity in treatment effects on depressive symptoms in OSA. Additionally, we sought to examine the effect of MAD therapy on depressive symptoms.

## Methods

This study was performed using a prespecified protocol. All measures of reporting and assessment are based on the Preferred Reporting Items for Systematic Reviews and Meta-analyses (PRISMA) statement [12].

### Inclusion/Exclusion Criteria

We included all randomized controlled trials (RCTs) of CPAP or MADs performed in adults aged 18 y and older with OSA that also measured depressive symptoms. We included trials if they reported a diagnosis of sleep apnea based on sleep study results that showed an apnea hypopnea index (AHI), respiratory disturbance index (RDI), or oxygen desaturation index (ODI)>5 events/hour; trials that used only questionnaires for diagnosis of OSA were excluded. A diagnosis of depression at baseline was not required; however, included trials required measurement of depressive symptoms using a validated depression questionnaire. These included the Beck Depression Inventory (BDI) [13], the Hospital Anxiety and Depression Scale depression subscale (HADSd) [14], the Montgomery-Asberg Depression Rating Scale (MADRS) [15], the Profile of Mood States depression subscale (POMSd) [16], the Geriatric Depression Scale (GDS) [17], the Brief Symptom Inventory (BSI) [18], the Center for Epidemiologic Studies Depression Scale (CESD) [19], and two subscales of the Short Form-36 (SF-36): SF-36 mental component score (SF-36 MCS) and SF-36 mental health (SF-36 MH) [20]. Trials investigating populations with co-morbid bipolar disorder, schizophrenia, or other psychiatric diagnoses were excluded. We did not limit trials by publication language or date.

### Data Sources and Searches

We searched four online databases: Medline, EMBASE (Excerpta Medica Database), the Cochrane Central Registry of Controlled Trials, and PsycINFO from the date of inception of the databases up until August 15, 2014. We also searched abstracts from the previous three years of the Associated Professional Sleep Societies conferences, the American Thoracic Society conferences, and the American Psychiatric Association conferences. This was done using electronic word searching tools using the same key words applied to online databases. Finally, two individuals (M. P. and C. E. B.) searched the reference lists of identified relevant publications. Experts in the field were contacted regarding information concerning unpublished studies and ongoing trials.

We searched each online database using common terms related to three major themes as subject headings or keywords in the title or abstract. The list of specific search terms for each online database is presented in Table S1. The first theme included terms relating to the population of adults with OSA. The second theme was related to the intervention of either CPAP or MADs. The third theme was related to study design, and was limited to RCTs. The appropriate Cochrane Collaboration filters were used to search for RCTs within each of the specific databases [21]. Finally, we combined the results of the three search themes using the Boolean operator “and” to identify trials possessing all specified criteria. Terms relating to “depression” as an outcome measure were not included in order to maximize the sensitivity of the search and to ensure that all trials involving CPAP or MAD treatment in OSA were identified, including those where depression was a secondary outcome.

### Study Selection

Both individuals (M. P. and C. E. B.) initially screened all identified abstracts for further review by verifying that the trial contained original data and was relevant to the research question (i.e., involved CPAP or MAD treatment in OSA patients, included a measure of depression, and included a control group). The initial screen was broad in an effort to capture all relevant literature. From the initial abstract screening, articles were reviewed further if M. P. or C. E. B. decided that full-text review was necessary. For these full-text articles, eligibility for inclusion in the systematic review was considered if they addressed all elements of our research question. Trials were included in the meta-analysis if they reported mean change and standard deviation of the change in depression score or pre- and post-intervention depression scores in both the treatment and control groups, or if sufficient data were presented to calculate the mean change and standard deviation of the change in depression score. Missing standard deviations were calculated from *p*-values or *t*-scores or were imputed using correlation coefficients generated from studies of similar design and duration, according to methods described in the *Cochrane Handbook for Systematic Reviews of Interventions* [22]. Where insufficient data were presented, the corresponding authors were contacted for additional information.

### Data Extraction and Quality Assessment:

A data extraction form was used to collect information from each identified trial. Data was extracted in duplicate by the investigators (M. P. and C. E. B.), and disagreement was resolved by consensus. There were eight different depression scales (counting the two SF-36 subscales as one) used across studies, with several studies reporting multiple scales. The detail of reporting did not always allow for extraction of all included scales for each study, but where possible, all results were extracted for possible synthesis. The quality of each study was assessed based on components of the Cochrane risk of bias tool [23].

### Data Synthesis and Analysis

The primary outcome measure was the mean difference between treatment and control groups in the change in depression scores from baseline to the end of the trial. In order to maintain a consistent direction of effect between different depression scales [22], we reversed the direction of change scores where necessary so that positive values corresponded to improvement in depression for all trials. Studies that compared three arms (placebo, CPAP, and MADs) were identified in the systematic review; however, only results from comparisons between CPAP and control or MADs and control were included in meta-analyses.

In order to compare results across trials that used different scales for depressive symptoms, we pooled the standardized mean difference (SMD) using the random effects model of DerSimonian and Laird [22]. We chose a random effects model to generalize our results to the variety of populations studied with OSA. Analyses were performed separately based on treatment method (CPAP or MAD). For the purposes of the meta-analysis of the SMDs, we included each trial only once and preferentially included the scale used by each trial that was most specific to depression, rather than the SF-36 MH score, wherever possible. We performed random effects meta-analysis using the pooled weighted mean difference (WMD) if there were two or more trials that used a common depression scale. We performed stratified meta-analyses and meta-regression on seven different study-level characteristics that we specified a priori as potential sources of significant heterogeneity. These included RCT design type (parallel or crossover), OSA severity (mild to moderate or severe), blinding of both patients and assessors (yes or no), trial length (<4 wk, 4–8 wk, or 8+ wk), mean treatment adherence (<4 h/night or 4+ h/night), baseline depression based on established questionnaire cutoff values (SF-36 MCS, <42; SF-36 MH, <52; BDI, >14; HADSd, >8; BSI depression subscale, >0.28; POMSd, >23) [16],[18],[24]–[28], and type of depression measurement tool (SF-36 versus other). We considered an alpha = 0.10 as significant in meta-regression [29].

Funnel plots and Begg's test were used to assess for small study effects indicative of publication bias. STATA IC (STATACorp College Station, Texas, US) was used for all statistical analysis, using the metan, metareg, metafunnel, heterogi, and metabias commands to generate pooled estimates and plots.

## Results

### Study Identification and Characteristics

**Figure 1:**
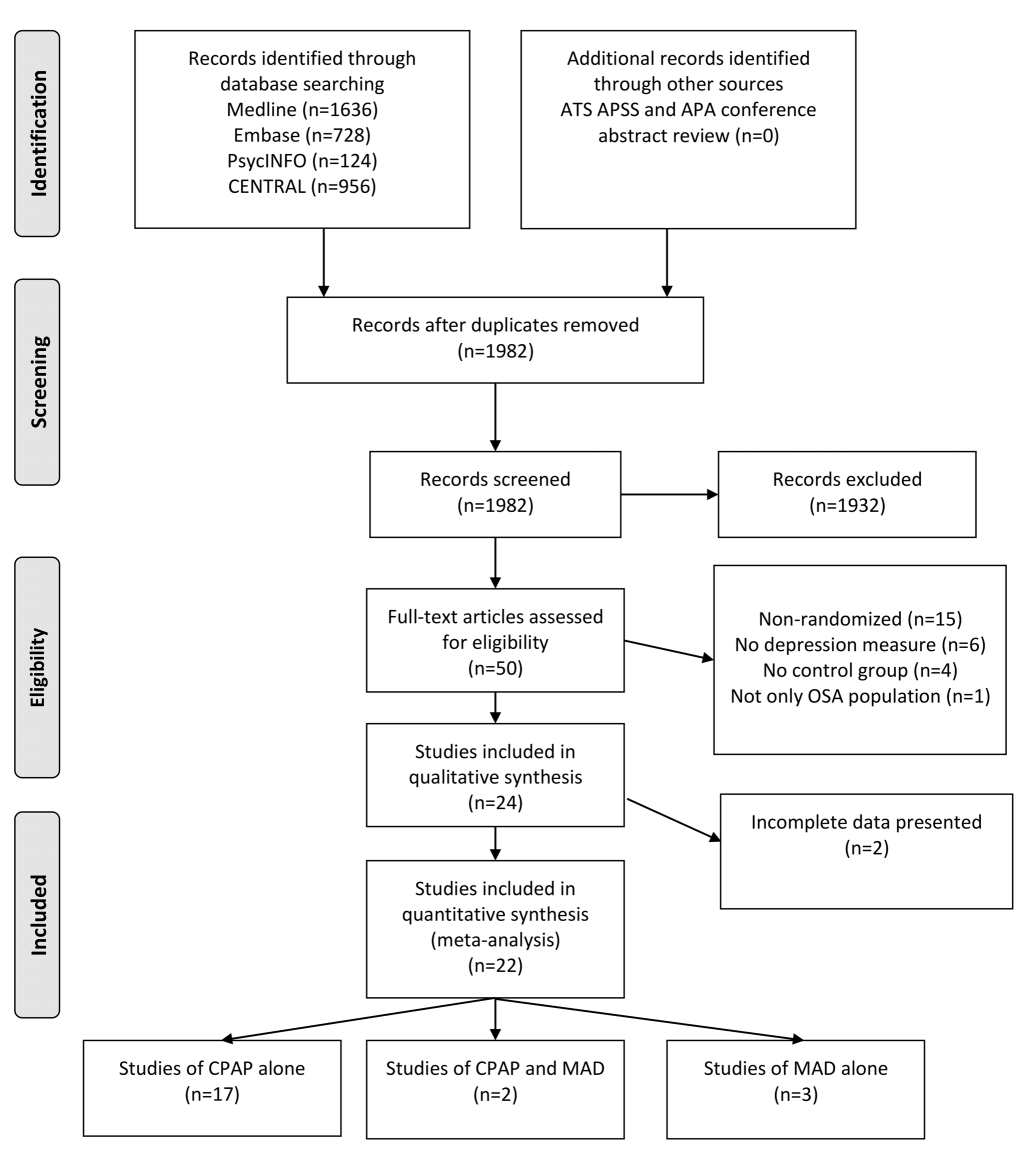
PRISMA flow diagram [12]. All medical databases were searched on the same date. Abstract, full-text review, and data extraction were conducted in duplicate by M. P. and C. E. B. APA, American Psychiatric Association; ATS, American Thoracic Society; APSS, Associated Professional Sleep Societies; CENTRAL, Cochrane Central Registry of Controlled Trials.

Our systematic search identified 1,982 unique citations. No additional citations were discovered from conference proceedings. Of the 1,982 articles, 50 were selected for full-text review. Our full-text review identified 24 RCTs, including 26 randomized comparisons evaluating the change in depressive symptoms from baseline to study end in response to either CPAP or MAD therapy in OSA patients (Figure 1). The kappa statistic for agreement between the two reviewers for inclusion was excellent: κ = 0.87 (95% CI: 0.72–0.99).

All 24 RCTs identified were full-text articles published in peer-reviewed journals between 1998 and 2014 [30]–[53]. Eleven of the studies were published since the last systematic review on this topic was conducted [7]. Fifteen of the RCTs were parallel trials, while seven were crossover trials. Two trials had elements of both designs, but reported results at the end of the first treatment period, and therefore for the purposes of meta-analysis, the crossover component was ignored. Details of the study-level characteristics of all included trials are presented in Table 1. Six studies excluded individuals using antidepressant medications, and one study excluded individuals using benzodiazepines; the remainder of the studies did not report use of anti-depressant medications nor describe exclusion criteria based on their use. None of the studies reported initiation of antidepressant use during the study period. The mean age of participants ranged from 42 to 78 y, the proportion of men ranged from 46% to 100%, and the mean body mass index ranged from 24.7 to 42.5 kg/m^2^. There were 19 trials of CPAP treatment, three of MADs, and two trials of both treatments. The most common type of control was sham CPAP or sham MAD, although an oral placebo tablet, sham exercises, or no therapy was also used as a comparator (Table 1). The OSA severity of the study populations ranged from mild to severe, with mean RDI/ODI/AHI of 9.8–62.4 events/hour. Treatment length was from 1 to 24 wk, while mean adherence to treatment ranged from 2.3 to 7.7 h/night.

**Table 1:** Characteristics of included studies on the effect of CPAP or MADs on depression in patients with OSA.

Study	Number of Participants	Mean Age (Years)/Percent Male	Mean AHI (Events/h)+	OSA Treatment Method	Mean Adherence in Treatment Arm (h/Night)	Control	Mean Adherence in Control Arm (h/Night or Percent of Nights)	Depression Scale(s) (Mean Score)	Length of Trial (Weeks)
Amin et al. 2011 [30]	18	42/100	19	CPAP	4.4	Sham CPAP	4.4	SF-36 MCS (32.4)	3
Bardwell et al. 2007* # [31]	38	45.9/87	RDI: 62.3	CPAP	6.3	Sham CPAP	6.0	BSI (0.29)	2
Barnes et al. 2002 [32]	42	45.5/83	12.9	CPAP	3.5	Oral placebo	93%	BDI, SF-36 MH (72.5)	8
Barnes et al. 2004 [33]	114	47/80	21.3	CPAP and MAD	CPAP: 3.6; MAD: 5.5	Oral placebo	94%	BDI (9.2)	12
Blanco et al. 2005 [34]	24	54.3/83	28.9	MAD	7.7	Sham MAD	6.5	SF-36 MH (60.1)	12
Craig et al. 2012 [35]	391	57.8/78	ODI: 9.8	CPAP	2.3	Standard care	NR	SF-36 MCS (48.2)	24
Diaferia et al. 2013 [36]	51	44.8/100	31.3	CPAP	3.6	Sham exercises	NR	SF-36 MH	12
Engleman et al. 1998 [37]	23	47/91	43	CPAP	3.2	Oral placebo	NR	HADSd (5.7)	4
Engleman et al. 1999 [38]	34	44/62	10	CPAP	3.2	Oral placebo	NR	HADSd (7.4), SF-36	4
Haensel et al. 2007 [39]	50	48.6/80	61	CPAP	6.6	Sham CPAP	6	POMSd (7.2)	2
Henke et al. 2001 [40]	46	50.4/54	65.1	CPAP	5.9	Sham CPAP	5.2	GDS	1.9–5.4
Jenkinson et al. 1999* [41]	101	49&/100	ODI: 30.7	CPAP	5.4	Sham CPAP	4.6	SF-36 MH (71)	4
Lam et al. 2007 [42]	101	46/78	21.4	CPAP and MAD	CPAP: 4.2; MAD: 6.4	Standard Care	NR	SF-36 (12.6)	10
Lee et al. 2012* # [43]	71	48.3/66	34	CPAP	NR	Sham CPAP	NR	POMSd (12.5), CESD, BSI	3
Marshall et al. 2005# [44]	31	50.5/76	21.6	CPAP	4.9	Sham CPAP	4.9	HADS (4.2), SF-36	3
Montserrat et al. 2001 [45]	48	54.2/91	53.8	CPAP	4.3	Sham CPAP	4.5	SF-36 MH (71.83)	6
Naismith et al. 2005# [46]	86	48.4/81	26.9	MAD	6.7	Sham MAD	6.7	BDI (5.8), POMSd	4
Petri et al. 2008 [47]	93	50/83	34.7	MAD	NR	Sham MAD	NR	SF-36 MH (71)	4
Redline et al. 1998$ [48]	97	48.7/52	RDI: 13.1	CPAP	3.1	Standard care	82%	POMSd (7.3)	10
Ryan et al. 2011 [49]	48	61.8/79	35.9	CPAP	5.0	Standard care	NR	BDI (5.8)	4
Sandberg et al. 2001 [50]	63	77.5/46	28	CPAP	4.1	Standard care	NR	MADRS (21)	4
Siccoli et al. 2008 [51]	102	48.4/100	ODI: 42.3	CPAP	4.7	Sham CPAP	3.9	SF-36 MH (72.6)	4
Smith et al. 2007 [52]	26	61/88	36	CPAP	3.5	Sham CPAP	3.3	SF-36 MH (78)	6
Yu et al. 1999* $ [53]	34	48.2/76	RDI: 40.6	CPAP	5.6	Sham CPAP	5.2	POMSd (12.5)	1

Unless otherwise indicated, studies did not report medication or antidepressant use at baseline. No studies reported whether antidepressants were initiated during the course of the study. Cutoffs for depression scales: SF-36 MCS, <42; SF-36 MH, <52; BSI depression subscale, >0.28; BDI, >14; HADSd, >8, POMSd, >2.

+ For ODI, the events were oxygen desaturations of 4% or greater.

*Depression was a primary outcome.

# Study excluded use of psychotropics including antidepressants at baseline.

& Median age.

$ Excluded use of benzodiazepines but not antidepressants.

NR, not reported.

### Risk of Bias

The quality of included trials varied (Tables 2 and 3). Almost all trials reported inclusion and exclusion criteria; however, only 56% of the trials reported the method of randomization, and allocation concealment was not reported or not performed in 48% of cases. Approximately half of the trials blinded both patients and assessors. Baseline differences between the treatment and control groups were infrequent. The majority of trials reported on loss to follow-up, and more than half of the trials performed a power calculation (Tables 2 and 3). For the crossover trials, the washout period ranged from 7 to 28 d, with two trials having no washout period (Table 3).

**Table 2:** Assessment of bias of included parallel RCTs on the effect of CPAP or MAD on depression in patients with OSA.

Study	Inclusion/Exclusion Specified	Method of Randomization Given	Allocation Concealment	Patients Blinded	Assessors Blinded	Reported Loss to Follow-Up	Baseline Difference between Groups	Power Calculation	Intention to Treat Analysis	Funding Source Disclosed
Amin et al. 2011 [30]	Yes	No	NR	Yes	No	Yes	Yes	No	No	Yes
Bardwell et al. 2007 [31]	Yes	No	NR	Yes	Yes	No	No	Yes	No	No
Blanco et al. 2005 [34]	Yes	No	NR	Yes	NR	Yes	No	No	No	No
Craig et al. 2012 [35]	Yes	Yes	Yes	No	Yes	Yes	No	Yes	Yes	Yes
Diaferia et al. 2013 [36]	Yes	No	NR	No	No	Yes	No	No	No	Yes
Haensel et al. 2007 [39]	Yes	No	NR	Yes	Yes	No	No	No	No	Yes
Henke et al. 2001* [40]	Yes	No	NR	Yes	Yes	Yes	No	No	No	Yes
Jenkinson et al. 1999 [41]	Yes	Yes	Yes	Yes	Yes	Yes	No	Yes	No	Yes
Lam et al. 2007 [42]	Yes	No	NR	NR	NR	Yes	No	Yes	Yes	Yes
Lee et al. 2012 [43]	Yes	Yes	Yes	Yes	Yes	Yes	No	Yes	No	Yes
Montserrat et al. 2001* [45]	Yes	Yes	Yes	Yes	Yes	Yes	Yes	Yes	No	Yes
Petri et al. 2008 [47]	Yes	Yes	Yes	Yes	Yes	Yes	No	Yes	Yes	Yes
Redline et al. 1998 [48]	Yes	Yes	No	No	NR	Yes	Yes	No	NR	Yes
Ryan et al. 2011 [49]	Yes	Yes	Yes	No	Yes	Yes	No	Yes	No	Yes
Sandberg et al. 2001 [50]	No	Yes	Yes	No	NR	Yes	No	No	No	Yes
Siccoli et al. 2008 [51]	Yes	No	NR	Yes	Yes	Yes	No	No	Yes	Yes
Yu et al. 1999 [53]	Yes	No	NR	Yes	NR	Yes	Yes	No	No	Yes

*Crossover period excluded.

NR, not reported or unclear.

**Table 3:** Assessment of bias of included crossover RCTs on the effect of CPAP or MADs on depression in patients with OSA.

Study	Washout Period (Days)	Inclusion/Exclusion Specified	Method of Randomization Given	Allocation Concealment	Patients Blinded	Assessors Blinded	Reported Loss to Follow-Up	Baseline Difference between Groups	Power Calculation	Intention to Treat Analysis	Funding Source Disclosed
Barnes et al. 2004 [32]	14	Yes	Yes	Yes	No	Yes	Yes	No	Yes	Yes	Yes
Barnes et al. 2002 [33]	0	Yes	Yes	Yes	Yes	Yes	Yes	No	Yes	No	Yes
Engleman et al. 1999 [37]	0	Yes	Yes	NR	NR	NR	Yes	No	Yes	No	Yes
Engleman et al. 1998 [38]	28	Yes	No	NR	NR	NR	Yes	No	No	No	Yes
Marshall et al. 2005 [44]	14	Yes	Yes	Yes	Yes	Yes	Yes	No	Yes	No	Yes
Naismith et al. 2005 [46]	7	Yes	Yes	Yes	Yes	Yes	Yes	No	No	No	Yes
Smith et al. 2007 [52]	7	Yes	Yes	Yes	Yes	Yes	Yes	No	Yes	No	Yes

NR, not reported or unclear.

### Efficacy of CPAP for Depression

**Figure 2:**
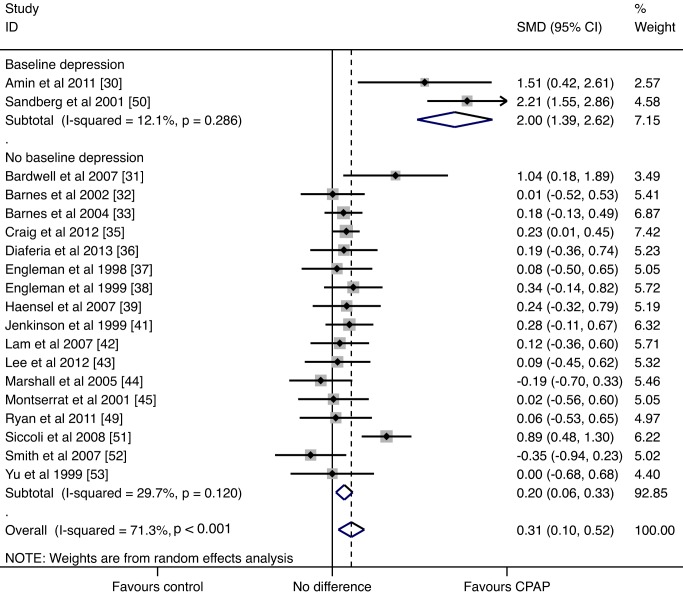
CPAP studies forest plot. Data were calculated by a random effects model. Studies were stratified by baseline depression score. Boxes are SMDs, and lines are 95% CIs. The vertical solid line represents no difference between CPAP and control. Values to the right of the solid line favor CPAP benefit. Pooled SMDs and 95% CIs are represented by the diamond shapes.

We identified 21 trials that reported the effect of CPAP on depression symptoms, and 19 trials (1,355 participants in total) provided sufficient data for meta-analysis. The two excluded trials [40],[48] did not provide results of depression questionnaire testing, though it was described in the methods. Supplemental information for these trials could not be obtained. In random effects meta-analysis, CPAP treatment resulted in an improvement in depressive symptoms compared to the control group: SMD = 0.312 (95% CI: 0.099, 0.525) (*p* = 0.004) (Figure 2). There was significant heterogeneity between the trials (*Q* statistic, *p*<0.001; *I* ^2^ = 71.3%, 95% CI: 54%, 82%), suggesting that much of the total variability in the pooled SMD could be attributed to between-trial heterogeneity.

The results of stratified analyses and meta-regression to explore reasons for the observed heterogeneity in the CPAP trials based on the seven study-level factors are presented in Table 4. Only two of the 21 comparisons included study populations with average baseline depression scores that met the depression cutoff according to the particular depression scale used (Table 1) [16],[18],[24]–[28]. The treatment effect of CPAP was significantly larger in populations whose mean baseline depression scores were above the cutoff for depression. The pooled SMD from trials of study populations with baseline depression was 2.004 (95% CI: 1.387, 2.621), compared with 0.197 (95% CI: 0.059, 0.334) for study populations with baseline depression scores under the cutoff for depression (meta-regression, *p*<0.001) (Figure 2). In a sensitivity analysis in which the trial by Bardwell et al., which was close to the cutoff value, was re-stratified into the depressed stratum, similar results were found (SMD = 1.635, 95% CI: 0.880, 2.391). The treatment effect of CPAP was also modified by the type of RCT design, with a greater treatment effect observed in parallel arm trials (Figure S1). The pooled SMD for parallel arm RCTs was 0.506 (95% CI: 0.196, 0.817), compared to 0.063 (95% CI: −0.116, 0.243) for trials with crossover design (meta-regression, *p* = 0.076).

**Table 4:** Meta-regression analysis for effect of CPAP on depressive symptoms.

CPAP Treatment	Pooled SMD (95% CI)	*I* ^2^ Statistic (95% CI)	*p*-Value from Meta-Regression
**Overall**	0.312 (0.099, 0.525)	71 (54, 82)	—
**RCT design**			
Parallel	0.506 (0.196, 0.817)	78 (62, 87)	0.076
Crossover	0.063 (−0.116, 0.243)	0 (0, 71)	
**Depression measurement tool**			
SF-36 MH or SF-36 MCS	0.259 (0.010, 0.509)	61 (19, 81)	0.725
Other depression scale	0.373 (0.001, 0.745)	79 (61, 88)	
**OSA severity** *			
Mild to moderate	0.457 (0.063, 0.850)	84 (70, 91)	0.408
Severe	0.228 (−0.002, 0.458)	48 (0, 74)	
**Blinding**			
No	0.389 (0.093, 0.686)	76 (57, 87)	0.508
Yes	0.214 (−0.112, 0.540)	67 (29, 84)	
**Trial length**			
<4 wk	0.310 (−0.107, 0.728)	58 (0, 83)	0.708
4–8 wk	0.385 (−0.038, 0.807)	83 (70, 91)	
8+ wk	0.202 (0.042, 0.363)	0 (0, 85)	
**Mean adherence**			
<4 h/night	0.472 (0.131, 0.813)	80 (65, 88)	0.213
4+ h/night	0.161 (−0.001, 0.323)	0 (0, 75)	
Not reported	0.086 (−0.451, 0.623)	—	
**Baseline depression**			
No	0.197 (0.059, 0.334)	30 (0, 61)	<0.001
Yes	2.004 (1.387, 2.621)	12 (—)	

Meta-regression was performed to explore heterogeneity of pooled SMDs in depression scores for CPAP treatment versus control. Positive values indicate a greater improvement in depression symptoms with CPAP treatment than control. *I* ^2^ statistic provides a measure of heterogeneity, with lower values indicating less heterogeneity. *p*-Values were considered significant with an alpha of 0.10. Dashes indicate values that could not be calculated.

*Mild to moderate OSA defined as AHI/RDI <30 or ODI<20. Severe OSA defined as AHI/RDI>30 or ODI>20.

The pooled analyses of the WMD in depression score with CPAP treatment versus control for the three most common depression scales are presented in Table 5. Pooled results for subscales of the SF-36, POMSd, and BDI all showed improvements in depressive symptoms with CPAP treatment, although these estimates were not statistically significant. When the SF-36 MCS trials were stratified by the presence or absence of depression at baseline in the trial populations, the trial with a population with depression symptoms at baseline showed a significant improvement in SF-36 MCS score, with a WMD of 10.800 (95% CI: 3.981, 17.619) for the CPAP versus control arm of the trial.

**Table 5:** Pooled weighted mean differences in depression score for CPAP treatment.

Depression Measure	Number of Trials	Pooled WMD (95% CI)	*I* ^2^ Statistic (95% CI)
SF-36 MH	9	1.914 (−1.366, 5.194)	57 (10, 80)
SF-36 MCS—baseline depression	1	10.800 (3.981, 17.619)	—
SF-36 MCS—no baseline depression	5	2.039 (−1.710, 5.789)	77 (43, 90)
POMSd	3	0.581 (−1.253, 2.415)	0 (0,90)
BDI	2	0.997 (−0.666, 2.660)	0 (—)

WMDs were calculated for studies using the same measure of depressive symptoms. The most common depression scales were included in this analysis. The pooled estimated WMD is in the units of the scale used. Positive values indicate that a benefit of CPAP was seen. Dashes indicate values that could not be calculated.

After removal of the studies with baseline depression symptoms that introduced heterogeneity, analysis of the funnel plot did not show asymmetry (Figure S2). Begg's test was non-significant (*p* = 0.317), suggesting that there was no evidence of small study effects that would suggest publication bias.

### Efficacy of MADs for Depression

**Figure 3:**
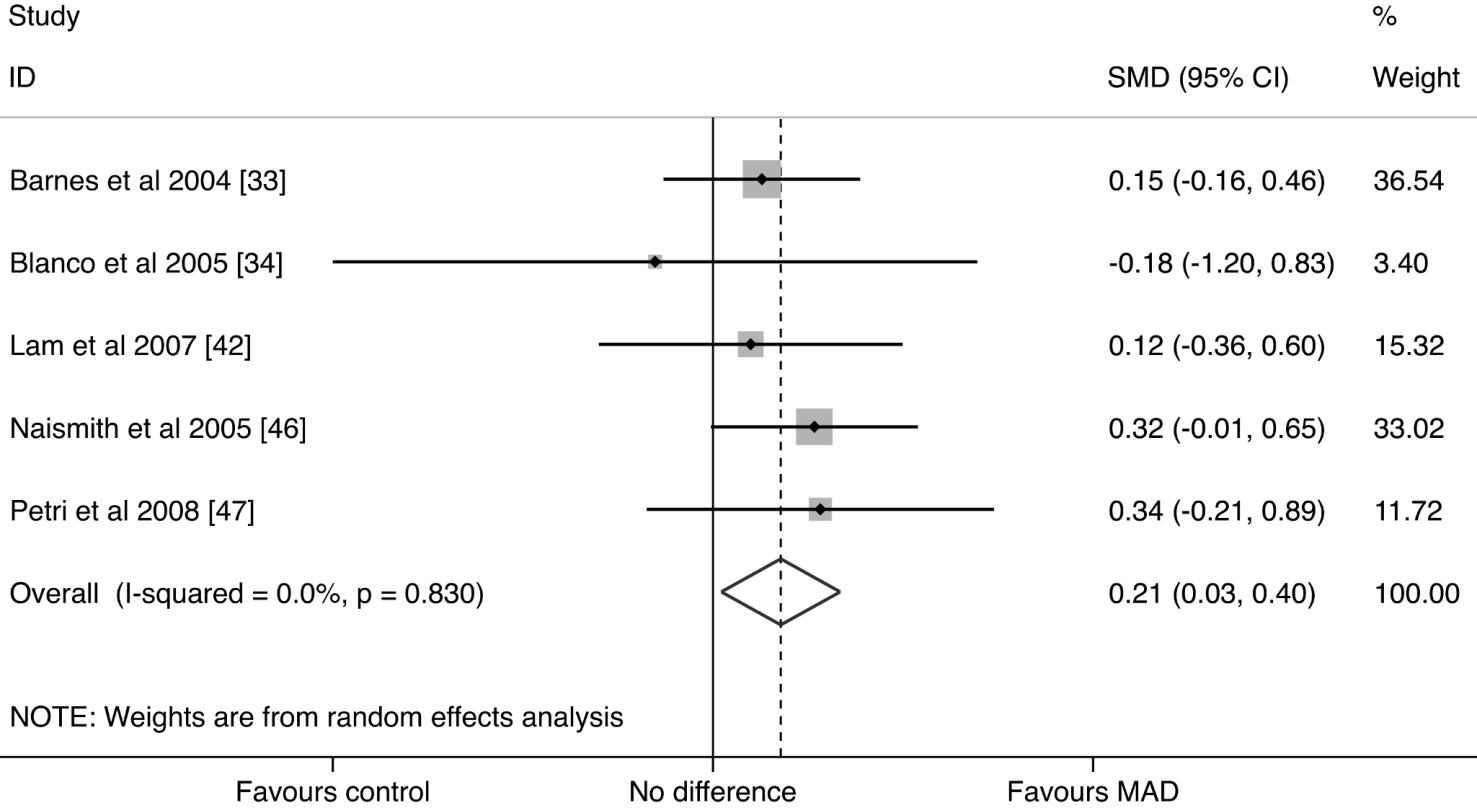
MAD studies forest plot. Data were calculated by a random effects model. Boxes are SMDs, and lines are 95% CIs. The vertical solid line represents no difference between MAD and control. Values to the right of the solid line favor MAD benefit. Pooled SMD and 95% CI is represented by the diamond shape.

We identified five trials that reported the effect of MADs on depressive symptoms (440 participants total). Random effects meta-analysis of these five trials of MADs showed a significant improvement in depressive symptoms with MAD treatment compared to controls: SMD = 0.214 (95% CI: 0.026, 0.401) (*p* = 0.025) (Figure 3). The associated *Q* statistic did not indicate significant heterogeneity (*p* = 0.830), and *I* ^2^ was 0% (95% CI: 0%, 79%), indicating that the observed variability was due to chance or sampling error. The WMD for the three trials using the SF-36 MH subscale was 2.984 (95% CI: −1.812, 7.780), and the WMD for the two trials using the BDI was 0.800 (95% CI: 0.076, 1.524) (Table 6). These WMDs illustrated improvements in depressive symptoms with MADs, although only the WMD for the BDI scale achieved statistical significance.

**Table 6:** Pooled weighted mean differences in depression score for MAD treatment.

Depression Measure	Number of Trials	Pooled WMD (95% CI)	*I* ^2^ Statistic (95% CI)
SF-36 MH	3	2.984 (−1.812, 7.780)	0 (0, 90)
BDI	2	0.800 (0.076, 1.524)	0 (—)

WMDs were calculated for studies using the same measure of depressive symptoms. The pooled estimated WMD is in the units of the scale used. Positive values indicate that a benefit of MAD treatment was seen. The most common depression scales were included in this analysis. Dashes indicate values that could not be calculated.

## Discussion

In this meta-analysis of 22 RCTs—17 including CPAP therapy, three including MAD therapy, and two including both types of therapy—we found a modest benefit of CPAP treatment for improving depressive symptoms in individuals with OSA compared to control. The overall effect size found was representative of a change on the SF-36 MH subscale of +3.12 units [20], while on the BDI this would represent a change of approximately −2.15 units [24],[54]. When we stratified trials based on the presence of depression symptoms at baseline, we observed treatment effects representative of +20 units on the SF-36 or −13.8 units on the BDI. As a five-unit change on the BDI scale represents a minimally important clinical difference [55], our results indicate that CPAP treatment in populations with depressive symptoms results in a clinically relevant improvement in depressive symptoms. For study populations with less burden of depressive symptoms, the clinical significance of the changes appears limited (+1.97 units on the SF-36 or −1.36 units on the BDI) and below the threshold of a clinically significant change. We also found that treatment with MADs resulted in improvement in depressive symptoms, with an improvement of +2.1 units on the the SF-36 or −1.6 units on the BDI compared to control. The severity of depression seen in these studies is similar to what might be seen overall in an unselected population referred for OSA testing [5],[56].

Our study provides new information on the efficacy of CPAP and MADs for treatment of depressive symptoms in OSA, including results from 11 trials published since the last systematic review of OSA treatment and resolving the unexplained variability in treatment effect reported in the prior systematic review [7]. In contrast to the previous systematic review of OSA therapy, which was limited to an analysis of studies using the HADSd, we found a statistically significant effect of treatment on depression symptoms when all studies and depression scales were included. We found that the majority of the heterogeneity seen in the strength of the treatment effect arose from the presence or not of depression at the start of treatment in the population studied. Additionally, we found that study design was an important modifier of the treatment effect, with a significant effect identified with use of a parallel arm design but not with use of a crossover design. The attenuation of the treatment effect in crossover trials may relate to insufficient time for recurrence of depressive symptoms between treatment periods, as many trials employed a short or no washout period.

There are limitations to our systematic review. These include methodological limitations within component trials, which included lack of blinding of patients and outcome assessors (which could bias towards finding a benefit), short follow-up duration (which could bias towards the null), and variable adherence with CPAP in some trials (which could attenuate the treatment benefit). However, in stratified analyses and meta-regression, we found that the presence or absence of these features did not significantly modify the effect of CPAP treatment on depression. The completeness of reporting of these studies may also be a significant limitation. We were able to calculate point estimates and variance estimates for treatment effects according to recommended methods for most trials; however, two trials of CPAP provided insufficient data for inclusion in the meta-analysis. One reported that the result of CPAP treatment was non-significant but did not provide a numerical comparison [40], and the other did not report anything other than that the testing was done [57]. A further limitation is the quality of normative values for depression questionnaires. However, our findings from a sensitivity analysis in which we re-stratified into the group of trials with baseline depression one trial with a baseline depression score that fell just below the threshold for a depressed population suggest that this issue does not invalidate our findings. Moreover, the reporting of use of antidepressant medications in the component trials was incomplete. Only six trials excluded participants using antidepressants, and one excluded use of benzodiazepines, but the remainder did not report the use of antidepressant medications, and none reported whether antidepressants were initiated during the study period. If patients in the CPAP or MAD treatment groups were preferentially started on antidepressants, this could bias the trials towards showing a benefit with CPAP or MADs. Another limitation is that we only identified trials that used dimensional depression scales as opposed to structured or semi-structured diagnostic interviews to measure depression outcomes. These scales include questions assessing sleep quality; however, this usually comprises only one question on the scale. Without detailed patient response data, it is not possible to exclude the possibility that the improvement in depressive symptom score is due to improvement in sleep-related symptoms alone. However, the larger treatment effect on depressive symptoms seen among the two trials that included patients with a greater burden of depression at baseline suggests that improvement in sleepiness alone is unlikely to completely explain the changes in depression scores in these trials. Future trials in clinically depressed populations are needed, with attention to what specific symptoms of depression have improved with treatment of OSA.

In conclusion, this systematic review summarizes the available literature on OSA treatment, demonstrating that both CPAP and MAD treatment result in small improvements in depressive symptoms based on questionnaires. Our results illustrate that the greatest benefit of CPAP treatment on depressive symptoms may occur in populations with worse depression scores at baseline. Given the limitations of studies included in this analysis, additional high-quality RCTs would be helpful to confirm the finding that treatment of co-morbid OSA can improve symptoms in patients with clinically significant depression, in particular that diagnosed by standardized clinical interview. Additionally, a comparison of OSA therapy with depression treatments such as antidepressants or psychotherapy would elaborate on an appropriate treatment algorithm for patients with both OSA and depression.

## Supporting Information

Figure S1 **CPAP study forest plot stratified by study design.** Data were calculated using a random effects model. Studies were stratified by study design: parallel versus crossover. Boxes are SMDs, and lines are 95% CIs. The vertical solid line represents no difference between CPAP and control. Values to the right of the solid line favor CPAP benefit. Pooled SMDs and 95% CIs are represented by the diamond shapes.
(TIF)

Figure S2 **Funnel plot of published studies.** The standard error (s.e.) of the SMD is plotted versus the SMD. Larger studies cluster at the top of the pyramid, while smaller studies are in the outer lower areas. Balancing of points between the left and right of the solid line indicates the absence of significant publication bias. The trials with populations that had depression at baseline have been excluded.
(TIF)

Table S1 **Medline search strategy.** Detailed search terms and filters applied to generate our search. Analogous terms were used for each respective database.
(DOCX)

Text S1 **PRISMA checklist.**
(DOC)

## Abbreviations

AHI: apnea hypopnea index
BDI: Beck Depression Inventory
BSI: Brief Symptom Inventory
CESD: Center for Epidemiologic Studies Depression Scale
CPAP: continuous positive airway pressure
GDS: Geriatric Depression Scale
HADS: Hospital Anxiety and Depression Scale
HADSd: Hospital Anxiety and Depression Scale depression subscale
MAD: mandibular advancement device
MADRS: Montgomery-Asberg Depression Rating Scale
ODI: oxygen desaturation index
OSA: obstructive sleep apnea
POMSd: Profile of Mood States depression subscale
RCT: randomized controlled trial
RDI: respiratory disturbance index
SF-36: Short Form-36
SF-36 MCS: Short Form-36 mental component score
SF-36 MH: Short Form-36 mental health survey
SMD: standardized mean difference
WMD: weighted mean difference
