# Supplementary figures and images for: Effect of Treatment of Obstructive Sleep Apnea on Depressive Symptoms: Systematic Review and Meta-Analysis
Source: PLoS Med. 2014 Nov 25;11(11):e1001762. doi: 10.1371/journal.pmed.1001762 (PMC4244041; doi:10.1371/journal.pmed.1001762)

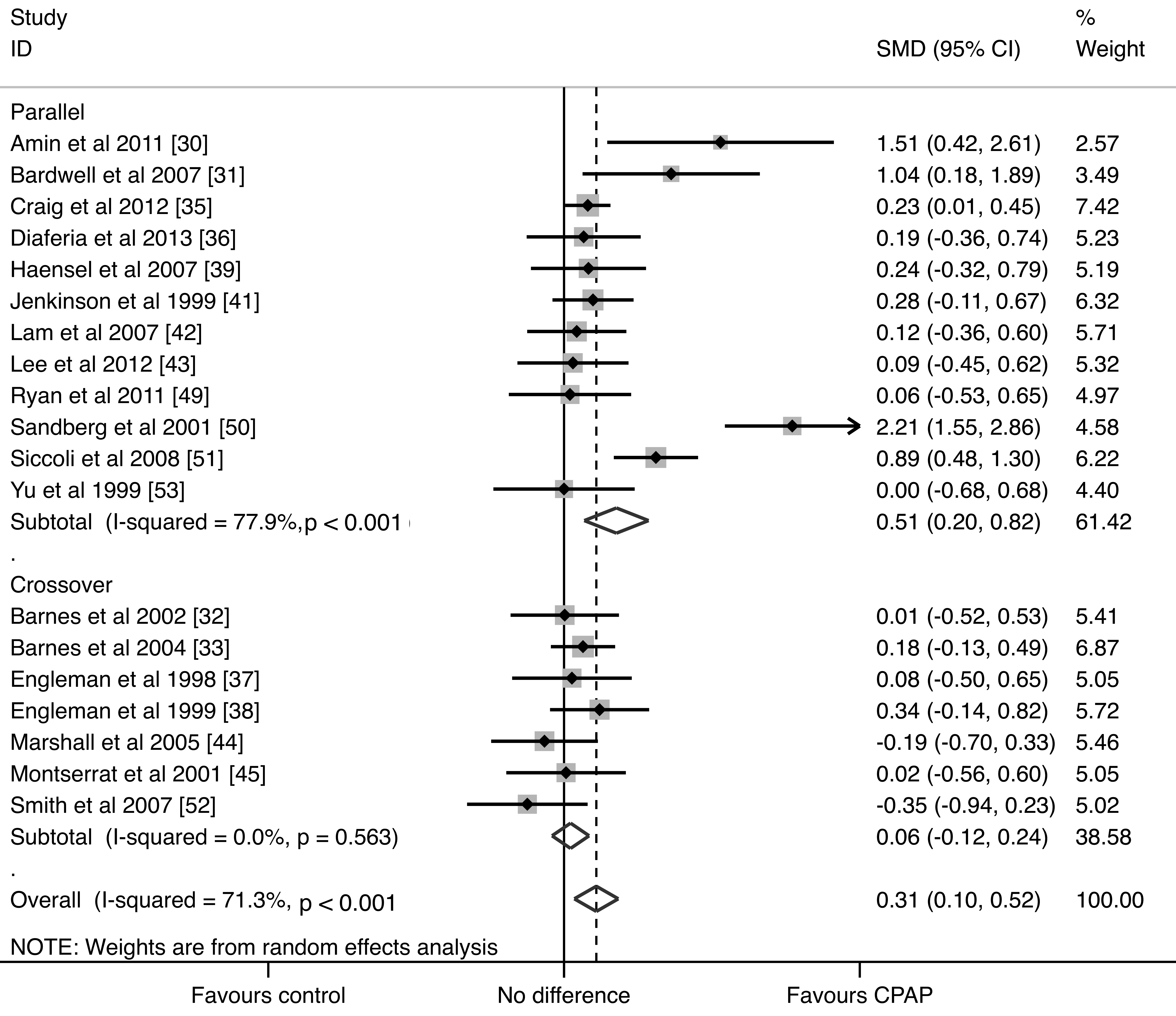

Supplement: Figure S1 — CPAP study forest plot stratified by study design. Data were calculated using a random effects model. Studies were stratified by study design: parallel versus crossover. Boxes are SMDs, and lines are 95% CIs. The vertical solid line represents no difference between CPAP and control. Values to the right of the solid line favor CPAP benefit. Pooled SMDs and 95% CIs are represented by the diamond shapes. (TIF) [file pmed.1001762.s001.tif]

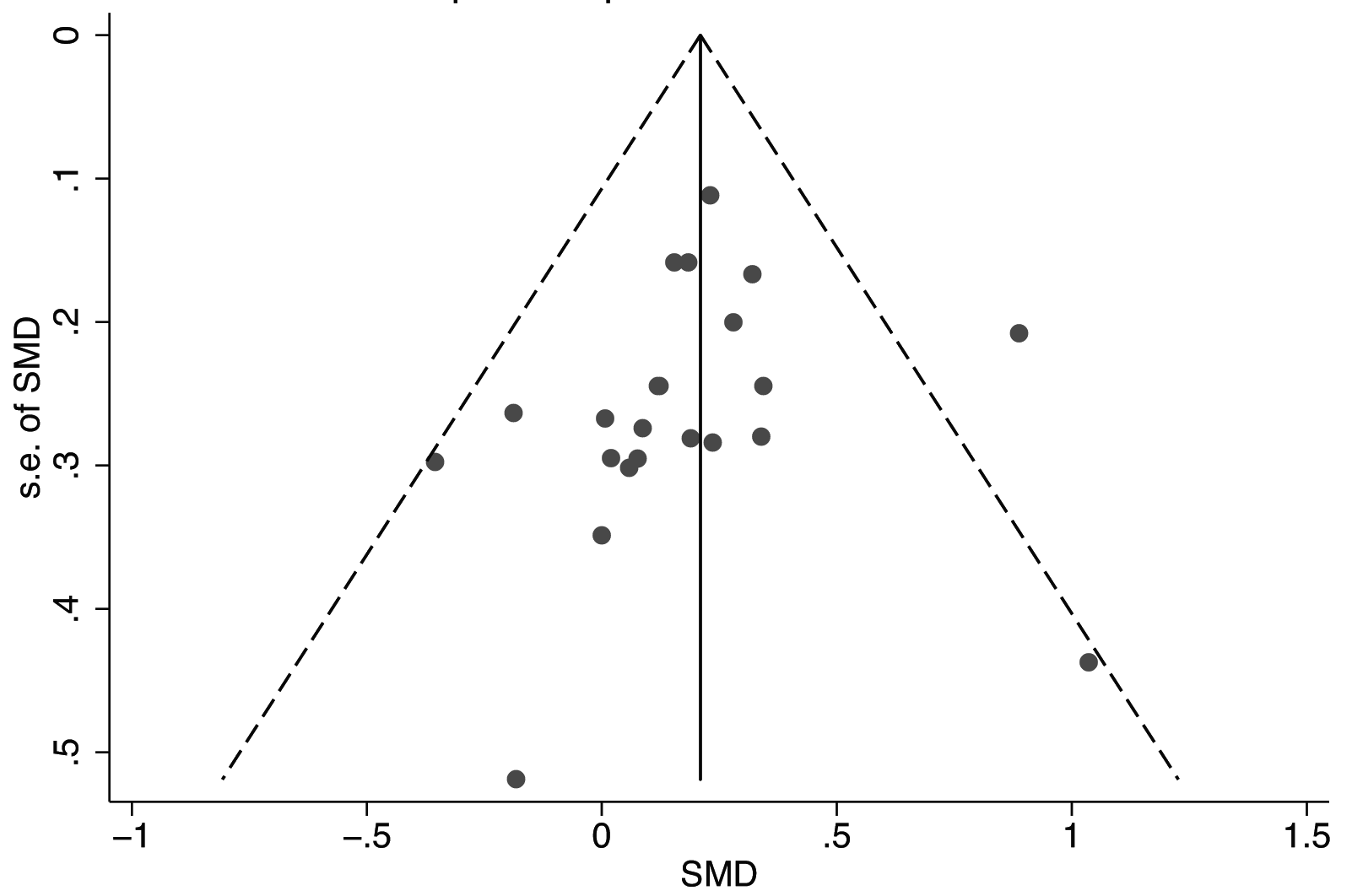

Supplement: Figure S2 — Funnel plot of published studies. The standard error (s.e.) of the SMD is plotted versus the SMD. Larger studies cluster at the top of the pyramid, while smaller studies are in the outer lower areas. Balancing of points between the left and right of the solid line indicates the absence of significant publication bias. The trials with populations that had depression at baseline have been excluded. (TIF) [file pmed.1001762.s002.tif]
