# Supplementary material for: Effect of Treatment of Obstructive Sleep Apnea on Depressive Symptoms: Systematic Review and Meta-Analysis
Source: PLoS Med. 2014 Nov 25;11(11):e1001762. doi: 10.1371/journal.pmed.1001762 (PMC4244041; doi:10.1371/journal.pmed.1001762)
Supplement: Table S1 — Medline search strategy. Detailed search terms and filters applied to generate our search. Analogous terms were used for each respective database. (DOCX) [file pmed.1001762.s003.docx]

|  | **Keywords** |
| --- | --- |
| 1 | Sleep Apnea, Obstructive/ |
| 2 | ("OSA" or "obstructive sleep apnea" or "sleep apnea" or "sleep disordered breathing").ab,ti. |
| 3 | Continuous Positive Airway Pressure/ |
| 4 | Mandibular Advancement/ |
| 5 | ("CPAP" or "continuous positive airway pressure" or "mandibular advancement device" or "dental appliance").ab,ti. |
| 6 | (controlled clinical trial or randomized controlled trial or meta analysis).pt. |
| 7 | (placebo* or random* or trial* or groups).tw. |
| 8 | 6 or 7 |
| 9 | exp animals/ not humans.sh. |
| 10 | 8 not 9 |
| 11 | 1 or 2 |
| 12 | 3 or 4 or 5 |
| 13 | 10 and 11 and 12 |
